# Clinical Characteristics Associated with High [^68^Ga]Ga-PSMA-11 Uptake and Preliminary Therapeutic Outcomes in Patients with Metastatic Adenoid Cystic Carcinoma

**DOI:** 10.1007/s13139-025-00955-9

**Published:** 2025-10-23

**Authors:** Hyunpil Sung, Minseok Suh, Joonhyung Gil, Bhumsuk Keam, Gi Jeong Cheon, Keon Wook Kang

**Affiliations:** 1 Department of Nuclear Medicine, Seoul National University Hospital, 101 Daehak-ro, Jongno-gu, Seoul, 03080 Republic of Korea; 2 Department of Molecular Medicine and Biopharmaceutical Sciences, Graduate School of Convergence Science and Technology, Seoul National University, Seoul, Republic of Korea; 3 Department of Internal Medicine, Seoul National University Hospital, 101 Daehak-ro, Jongno-gu, Seoul, 03080 Republic of Korea; 4 Cancer Research Institute, Seoul National University, Seoul, Republic of Korea

**Keywords:** Prostate-specific membrane antigen (PSMA), Adenoid cystic carcinoma (ACC), Theranostics, ^177^Lu-PSMA therapy

## Abstract

**Purpose:**

Adenoid cystic carcinoma (ACC) is a rare cancer that arises from the salivary glands. Recent studies have shown that prostate-specific membrane antigen (PSMA) expression is high in ACC. We conducted a prospective study to evaluate clinical factors associated with high [^68^Ga]Ga-PSMA-11 uptake and to report preliminary therapeutic outcomes of ^177^Lu-PSMA therapy in recurred or metastatic ACC.

**Materials and methods:**

Thirty patients were prospectively enrolled. Any focal accumulation of [^68^Ga]Ga-PSMA-11 not explained by physiological uptake was defined as pathological lesions. Clinicopathological features relevant for high [^68^Ga]Ga-PSMA-11 uptake in recurred or metastatic ACC were analyzed. Therapeutic outcomes of patients who received ^177^Lu-PSMA therapy under compassionate use were also reviewed as a secondary component.

**Results:**

A total of 238 lesions were evaluated, and the SUV_max_ ranged between 0.9 and 11.0, showing mild to moderate PSMA uptake. Higher SUV_max_ was observed in lung and bone metastases. The longest tumor diameter (*R* = 0.190, *P* = 0.009) and tumor % growth rate (*R* = 0.220, *P* = 0.006) were significantly correlated with SUV_max_. A longer duration from diagnosis was a relevant factor for high [^68^Ga]Ga-PSMA-11 uptake. Seven patients received ^177^Lu-PSMA therapy, but therapeutic outcomes were limited.

**Conclusion:**

High [^68^Ga]Ga-PSMA-11 uptake in recurred or metastatic ACC was associated with location of metastases, longer tumor diameter, high tumor % growth rate, and a longer duration from diagnosis. While ^177^Lu-PSMA therapy was performed in a limited cohort and outcomes were preliminary, these findings provide exploratory insights into potential factors relevant for patient selection and warrant further investigation in larger studies.

**Supplementary Information:**

The online version contains supplementary material available at 10.1007/s13139-025-00955-9.

## Introduction

Adenoid cystic carcinoma (ACC), a malignant tumor arising from the secretory glands, particularly the major and minor salivary glands, accounts for approximately 1% of all head and neck malignancies. ACC is a rare cancer with a reported incidence of 4.5 cases/per 100,000 individuals [1]. ACCs generally grow slowly; however, their long-term prognosis is poor owing to perineural invasion, multiple local recurrences, and high rates of distant metastases [2]. Complete surgical resection is the treatment of choice for ACC without distant metastasis. Radical radiation therapy can be applied to patients who are medically inoperable or have unresectable diseases. Standard treatment for ACC with distant metastasis has not been fully established [2, 3]. Recently, antiangiogenic agents have shown promising results for the treatment of recurrent and/or metastatic ACC [4].

Prostate-specific membrane antigen (PSMA) is a type II, 750-amino acid transmembrane protein that is anchored in the cell membrane of prostate epithelial cells. PSMA, which is significantly overexpressed in most prostate cancer cells, is attracting attention as a target molecule for theranostics [5]. When the PSMA-ligand is labeled with a β-emitting radionuclide, such as ^177^Lu, or an α-emitting radionuclide, such as ^225^Ac, a radioisotope-targeted therapy can be performed. In a recent clinical trial, ^177^Lu-PSMA radionuclide therapy improved imaging-based progression-free and overall survival when added to standard care in patients with metastatic castration-resistant prostate cancer [6]. [^177^Lu]Lu-PSMA-617 has now completed a phase 3 clinical trial and obtained FDA approval.

Previous studies have shown a high expression of PSMA in ACC [7–12]. In a retrospective review of ACC specimens, immunohistochemical expression of PSMA was observed in 94% of primary tumors [12]. Recent case series studies have demonstrated that high PSMA uptake is present in patients with recurred or metastatic ACC using ^68^Ga-labeled PSMA PET/CT [7, 9, 10]. In a standardized phase 2 clinical trial, [^68^Ga]Ga-PSMA-11 uptake higher than that in the liver was observed in 13 out of 14 (93%) patients with ACC. Overall, PSMA expression is reported to be high in ACC. When the PSMA ligand is labeled with a therapeutic isotope, it can be used as a new treatment for patients with ACC.

This prospective study was conducted to investigate clinical factors associated with high [^68^Ga]Ga-PSMA-11 uptake in patients with recurred or metastatic ACC, with preliminary therapeutic outcomes of ^177^Lu-PSMA treatment presented as ancillary findings.

## Materials and Methods

### Patients

Patients with recurrent or metastatic adenoid cystic carcinoma (ACC) were prospectively recruited between October 2021 and May 2022 for [^68^Ga]Ga-PSMA-11 PET/CT imaging. Between April 2022 and April 2023, patients received [^177^Lu]Lu-DOTA-Glu-Urea-Lys (DGUL) therapy under compassionate use approval; all but one patient in the treatment group had also been included in the imaging cohort. The study was approved by the Institutional Review Board (IRB no. H-2110-097-1263 for imaging; IRB no. H-2022-015-1297 for treatment), and all patients provided written informed consent.

### Image Acquisition

All patients underwent whole-body static PET/CT imaging 1 h after injection of [^68^Ga]Ga-PSMA-11 with a mean dose of 130 MBq. Emission scans were acquired over 15 min using dedicated PET/CT scanners (Biograph mCT 64, Siemens Medical Solutions, Forchheim, Germany), followed by CT scans for attenuation correction. PET images were reconstructed using an iterative algorithm (ordered-subset expectation maximization, OSEM).

### Treatment

[^177^Lu]Lu-DGUL is a radioligand for PSMA-directed therapy based on Glu-Urea-Lys derivatives [13]. The treatment plan involved administering 7.4 GBq of [^177^Lu]Lu-DGUL intravenously, every 6 weeks, for up to 6 cycles. Post-therapeutic whole body scan was performed two to three hours after administration. The eligible criteria for radioligand therapy was as follows: Patients over 19 years of age; patients with unresectable ACC with confirmed recurrence and metastasis; and patients showing positive results on PSMA PET. A positive PSMA result was defined as a highest tumor peak SUV (SUV_peak_) value greater than 1.5 times the average SUV (SUV_mean_) of the normal liver. Additionally, patients with adequate hematologic function, liver function, and renal function. The treatment responses were assessed according to RECIST 1.1 criteria in patients who completed at least two cycles of [^177^Lu]Lu-DGUL therapy, assessed 3 to 4 weeks after the last treatment.

### Image Analysis

Images were reviewed using MIM software (MIM Encore™, MIM Software Inc., Cleveland, OH, USA). Any focal accumulation of [^68^Ga]Ga-PSMA-11, not explained by physiological uptake was defined as a pathological lesion. The maximum standardized uptake value (SUV_max_) was measured for each lesion. For lung metastases, lesions with a longest diameter of ≥ 1 cm were evaluated, considering the partial volume effect and motion artifacts.

The SUV_mean_ was obtained for normal organs, including the salivary glands, liver, and blood pool. Volumes of interest (VOIs) were applied to the normal major salivary glands using a gradient-based segmentation method (PET edge). A spherical VOI with a 3 cm diameter was placed in the right hepatic lobe. For evaluation of the blood pool, circular regions of interest for the aorta were drawn on every 5-mm axial image and interpolated to acquire a single VOI, from the liver tip to the subcarinal level. There was no visual evidence of tumor involvement in any organ where the VOIs were placed.

The longest tumor diameter (mm) was measured and was measured on CT of PET/CT. The longest tumor diameter was also measured from immediately preceding the chest CT, if available. The tumor growth rate was defined as the percentage change in the longest tumor diameter per month (%/month). The median duration between PET/CT and preceding chest CT was 3.4 months (interquartile range [IQR]: 2.4–4.9 months).

### Statistical Analysis

Statistical analyses were performed using the PRISM (version 5.0; GraphPad Software, San Diego, CA, USA) and MedCalc statistical packages (version 14.8; MedCalc Statistical Software, Mariakerke, Belgium). For the lesion-based analysis, the differences in SUV_max_ according to the metastatic sites were analyzed using the Kruskal–Wallis test, followed by Dunn’s multiple comparison test for post-hoc analysis. The Pearson correlation coefficient was calculated to examine the correlation between SUV_max_ and CT-derived parameters (longest tumor diameter and tumor % growth rate). For the patient-based analysis, patients were categorized into high and low PSMA uptake groups, and their the clinicopathologic features were compared using the Fisher’s exact and Mann–Whitney U tests. A p value of less than 0.05 was considered to indicate a significant difference.

## Results

The patient characteristics are summarized in Supplementary Table 1. The median age of patients was 60.0 years (IQR: 50.3–66.0). In 14 patients, the primary tumor originated from the major salivary glands, and in 16 patients, from the minor salivary glands of the trachea, palate, tongue, paranasal sinus, and lip. All the patients underwent primary tumor resection and had distant metastases at the time of PSMA imaging. Out of a total seven patients, three received a single cycle of therapy, while the remaining four received two cycles.

### Lesion-Based Analysis

**Fig. 1:**
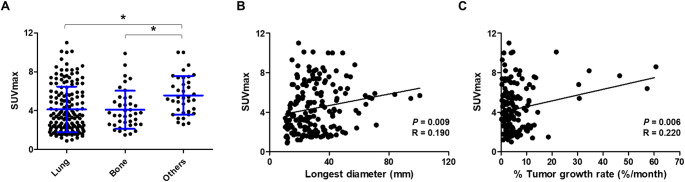
(**A**) The lesion SUV_max_ according to the metastatic sites was plotted and a groupwise comparison was conducted. The mean and the range of one standard deviation are indicated by blue guide lines. The correlation between SUV_max_ and (**B**) longest diameter of the lesions and (**C**) tumor % growth was plotted with Pearson correlation coefficient as R. (*, *p* < 0.05)

A total of 238 lesions were evaluated, with SUV_max_ ranging from 0.9 to 11.0 (mean ± standard deviation: 4.3 ± 2.3). Lung metastases were most common, followed by bone metastases. In addition, metastases to the pleura, liver, brain, lymph nodes, and soft tissues were observed. The mean ± standard deviation SUV_max_ was 4.14 ± 2.36 for lung lesions, 4.10 ± 1.97 for bone lesions, and 5.57 ± 1.97 for other metastatic sites. Lesion-based analysis showed a significant difference in SUV_max_ among metastatic sites (Kruskal–Wallis test, *p* = 0.005). Post-hoc pairwise comparisons using Dunn’s test with Bonferroni correction revealed that SUV_max_ was significantly higher in other metastatic sites compared with bone lesions (adjusted *p* = 0.004) and lung lesions (adjusted *p* = 0.047), whereas no significant difference was found between lung and bone lesions (adjusted *p* = 0.389). (Fig. 1A). The longest diameter of the metastatic lesions was measured in 185 lesions, and a weak, but significant correlation with lesion SUV_max_ was observed (*R* = 0.190, *p* = 0.009) (Fig. 1B). The tumor growth rate was measured in 153 lesions, and the median rate was 6.3%/month (± 8.7). The tumor growth rate showed a significant correlation with SUV_max_ (*R* = 0.220, *p* = 0.006) (Fig. 1C).

### Patient-Based Analysis

**Fig. 2:**
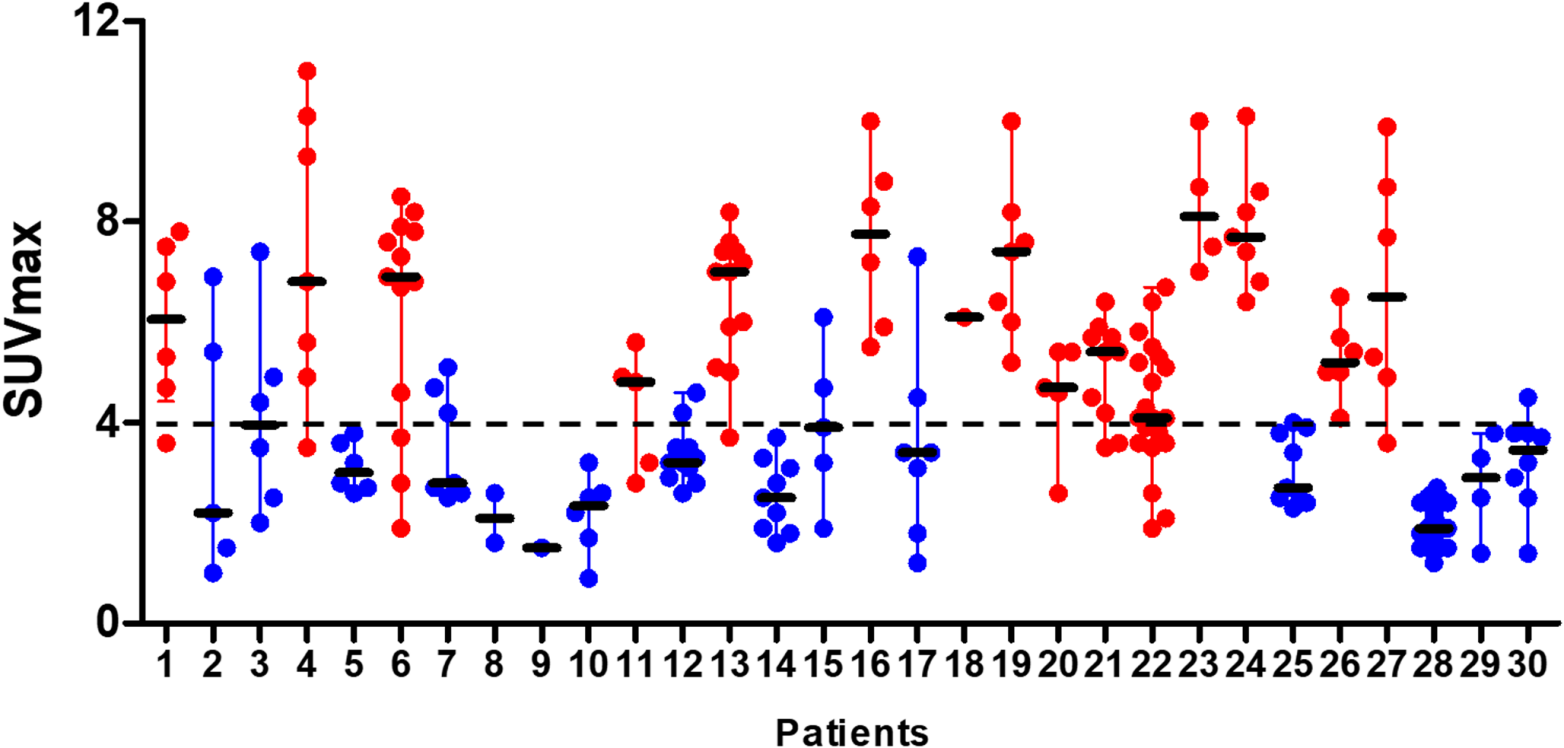
SUV_max_ of all lesions for each patient was plotted and inter- and intra-patient variations were observed. Patients were divided into high (red dots) and low (blue dots) PSMA uptake groups based on median lesion SUV_max_ of 4.0

Inter- and intra-patient variations were observed (Fig. 2). For the patient-based analysis median lesion, SUV_max_ was calculated for each patient, ranging form 1.5 to 8.1 (median, IQR: 4.0, 2.8–6.4). Patients were categorized into high and low PSMA uptake groups based on a median lesion SUV_max_ of 4.0. A comparison of the clinical characteristics between the two groups is summarized in Table 1. The duration from diagnosis to recurrence was longer in the high PSMA uptake group, but there was a statistically significant difference only in the duration from recurrence (43.4 vs. 19.6, respectively, *p* = 0.042). No significant differences were observed in age, sex, primary tumor location, predominant histological features, or previous history of chemotherapy. Furthermore, PSMA uptake in the liver, salivary glands, and blood pool was compared between the two groups, but no significant differences were observed (Supplementary Fig. 1).

**Table 1:** A comparison of the clinical characteristics between the high and low uptake groups

	High uptake	Low uptake	
Gender			
Male	7 (46.7%)	7 (46.7%)	*P* = 1.000
Female	8 (53.3%)	8 (53.3%)	
Age median (IQR)	56.0 (50.5–64.0)	63.0 (52.0–67.0)	*P* = 0.272
Primary tumor location			
Major salivary glands	6 (40%)	8 (53.3%)	*P* = 0.715
Minor salivary glands	9 (60%)	7 (46.7%)	
Predominant type			
Tubular or cribriform	5 (55.6%)	8 (88.9%)	*P* = 0.294
Solid	4 (44.4%)	1 (11.1%)	
Previous chemotherapy			
Done	7 (46.7%)	4 (26.7%)	*P* = 0.450
Not done	8 (53.3%)	11 (73.3%)	
Ongoing treatment			
Active surveillance	13 (86.7%)	13 (86.7%)	*P* = 1.000
Systemic therapy	2 (13.3%)	2 (13.3%)	
Duration median (IQR)			
from diagnosis (month)	76.9 (58.7–141.1)	55.1 (19.6–80.1)	*P* = 0.106
from recurrence (month)	43.4 (29.9–66.1)	19.6 (8.9–42.5)	*P* = 0.042

IQR, interquartile range

### Treatment

**Fig. 3:**
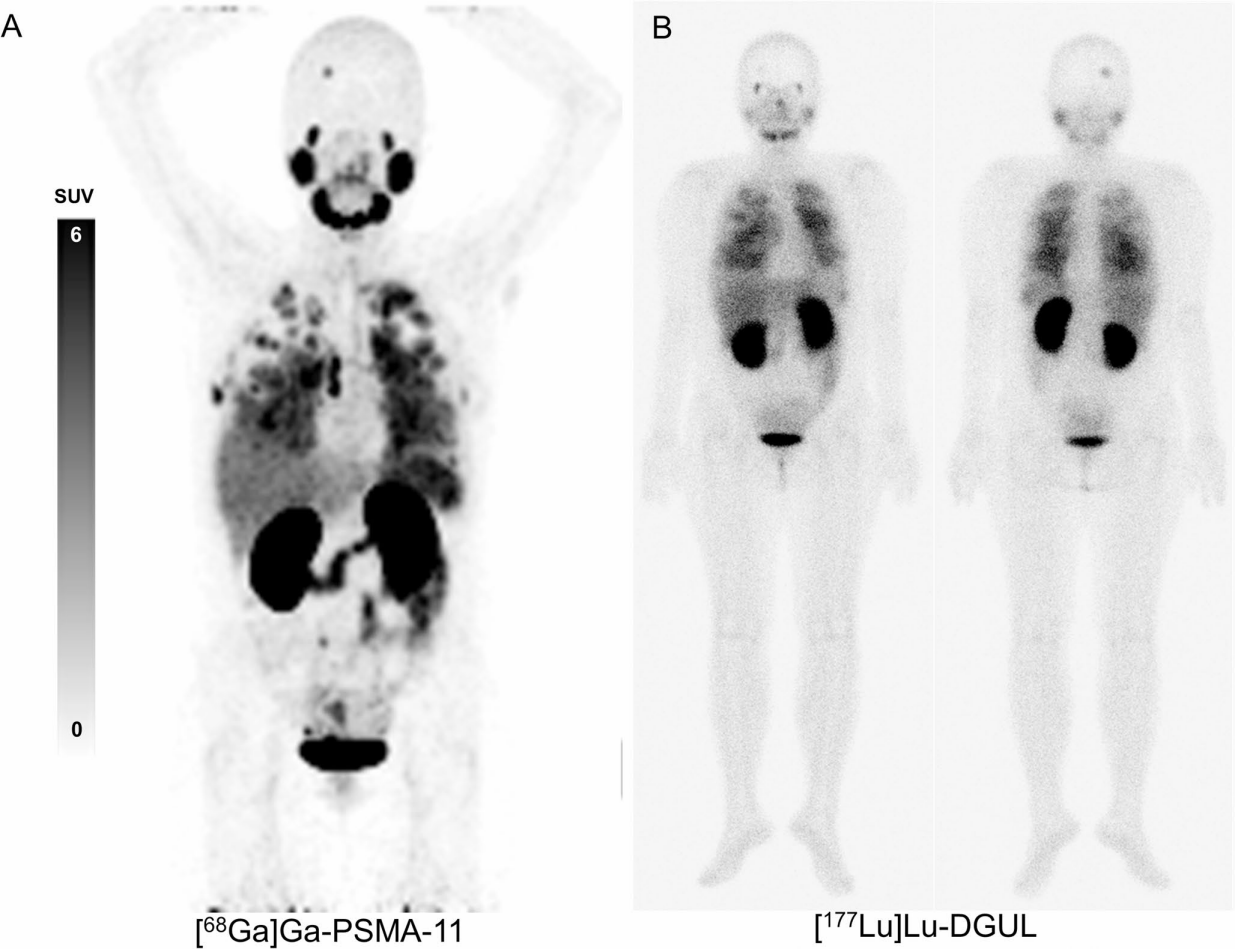
One patient (#21) was treated with [^177^Lu]Lu-DGUL, a novel PSMA targeting ligand based on Glu-Urea-Lys derivatives. (**A**) Baseline [^68^Ga]Ga-PSMA-11 scan and (**B**) [^177^Lu]Lu-DGUL Post-therapeutic scan showed a similar distribution in the lung, bone, brain metastases, and normal organs

The characteristics of patients who underwent [^177^Lu]Lu-DGUL therapy are summarized in Supplementary Table 2. No significant treatment-related adverse events occurred in the seven patients who underwent [^177^Lu]Lu-DGUL therapy. The treatment responses were assessed in four patients who completed two cycles of [^177^Lu]Lu-DGUL therapy. Two patients showed stable disease (SD), while the other two showed disease progression (PD). Even the two patients assessed as SD showed disease progression within a relative short time with new symptoms. One patient (#21) in the high PSMA uptake group was treated with [^177^Lu]Lu-DGUL. A similar distribution pattern of uptake in lung, bone, brain metastases, and normal organs was observed between 3-hour post-therapeutic [^177^Lu]Lu-DGUL and [^68^Ga]Ga-PSMA-11 images (Fig. 3).

## Discussion

We found that metastases other than those in the lung and bone, longer tumor diameter, and high tumor growth rate were associated with high [^68^Ga]Ga-PSMA-11 uptake in recurred or metastatic ACC. [^68^Ga]Ga-PSMA-11 uptake tended to be higher in patients with a longer diagnosis duration. Overall, more aggressive tumors demonstrated higher [^68^Ga]Ga-PSMA-11 uptake in recurred or metastatic ACC, which can be utilized for patient screening. However, the observed correlations with tumor size and growth rate were weak, and scatter plot analyses suggested that these relationships may not be strictly linear. These findings likely reflect underlying biological variability and heterogeneous tumor characteristics. Therefore, further studies using more detailed modeling approaches are warranted to better characterize these associations. The uptake mechanism of the PSMA imaging agent in ACC has not been fully elucidated. Unlike in prostate cancer, there was no significant correlation between the immunohistochemistry PSMA expression level and quantitative uptake of PSMA imaging agents in ACC [8, 11]. Similarly, a high accumulation of PSMA imaging agents in the salivary glands, from which ACCs originate, does not correspond to high PSMA expression levels, and the significant accumulation is due to off-target binding [14–16]. A thorough understanding of the PSMA uptake mechanism in ACC is required.

To date, various clinical trials of ^177^Lu-based PSMA therapy have been conducted in patients with prostate cancer. According to the PSMA-positive criteria from three clinical trials, the possibility of enrollment in ^177^Lu-based PSMA therapy was further evaluated (Table 2). In the VISION trial, when the uptake of metastatic lesions is higher than that of the liver and there are no PSMA-negative metastatic lesions, it is determined to be PSMA-positive [6]. Sixteen (53.3%) patients met the inclusion criteria. The TheraP trial used stricter criteria. A lesion must exceed an SUV_max_ of 20, and all other measurable metastatic lesions must exceed an SUV_max_ of 10 [17]. None of the patients in the study cohort met these criteria. In the LuPSMA trial, if the tumor SUV exceeded 1.5 × that of the liver SUV, it was determined as PSMA-positive [18]. Twenty-one (70%) patients met the criteria.

**Table 2:** The eligible patients in ^177^Lu-based PSMA therapy according to the PSMA-positive criteria from three clinical trials

	PSMA positive criteria	Eligible patients
VISION trial [6]	• Lesion PSMA uptake > liver • No PSMA-negative metastatic lesions • Bone with soft tissue component ≥ 1 cm • Lymph node ≥ 2.5 cm • Solid-organ ≥ 1 cm	16 (53.3%)
TheraP trial [16]	• SUV_max_ >20 at any site of disease • SUV_max_ >10 at all measurable sites	0
LuPSMA trial [17]	• SUV_max_ >1.5 times SUV of liver	21 (70.0%)

*PSMA* prostate-specific membrane antigen, *SUV *standardized uptake value

Compared with the SUV_max_ range of 1.1 to 30.2 in a previous study [11], the lesion SUV_max_ in patients with ACC in our study was generally lower. An international multicenter study revealed that tumor SUV, with time since the initial diagnosis of prostate cancer, chemotherapy status, and baseline hemoglobin concentration, was a predictor of ^177^Lu-PSMA therapy outcomes [19]. Seifert et al. reported that patients with prostate cancer with a low average PSMA uptake had significantly shorter survival, with a cutoff value of 14.3 [20]. Collectively, high levels of tumor PSMA expression are a prerequisite for favorable outcomes following ^177^Lu-PSMA therapy. Many studies have shown varying results regarding the efficacy of ^177^Lu-PSMA therapy. One study indicated that ^177^Lu-PSMA therapy may be clinically helpful in palliative settings [21]. This preliminary study included four patients with ACC, of which two (50%) were able to maintain stable disease and three (75%) showed immediate relief of tumor-related symptoms. On the other hand, another group reported limited efficacy of ^177^Lu-PSMA therapy for ACC and salivary duct carcinoma (SDC) [22]. This pilot study included ten ACC patients and six out of ten patients completed the four therapy cycles. However no objective responses were observed and there was a high screening failure rate. As demonstrated in our study, the therapeutic effect of ^177^Lu-PSMA therapy may be limited considering the relatively mild-to-moderate PSMA uptake in patients with ACC. Another possible explanation for the limited therapeutic outcomes is the treatment eligibility criterion adopted in this study. Specifically, although we required the highest tumor SUV_peak_ to be greater than 1.5 times the normal liver SUV_mean_, this criterion did not exclude the presence of PSMA-negative or low-uptake lesions within the same patient. As a result, heterogeneous uptake patterns may have contributed to suboptimal therapeutic efficacy. Given the limited treatment responses observed in a small number of patients, careful consideration in patient selection is essential. Since the difference between ACC and prostate cancer regarding the biological kinetics and radiation sensitivity is not fully understood, further validation is necessary to determine the treatment effect of ^177^Lu-PSMA therapy.

This study had some limitations. First, owing to the small sample size, we cannot make a generalized conclusion. Especially in treatment, all seven patients withdrew their consent before completing 4 cycles. However, to date, our study recruited the largest number of patients with ACC and is clinically relevant given the rarity of the disease. Secondly, there were patients without histopathology reports and tissue specimens; thus, comparison of pathological features and PSMA expression levels with [^68^Ga]Ga-PSMA-11 uptake was limited. Further studies with molecular and pathological information may elucidate the mechanism of PSMA uptake in patients with ACC.

## Conclusion

In patients with recurred or metastatic ACC, metastases other than those in the lung and bone, longer tumor diameter, higher tumor % growth rate, and a longer duration from diagnosis are relevant factors for high [^68^Ga]Ga-PSMA-11 uptake. Considering the relevant factors, candidates for ^177^Lu-PSMA therapy can be screened among patients with recurred or metastatic ACC. However, the actual therapeutic benefit of ^177^Lu-PSMA therapy remains limited. Given these preliminary therapeutic results, careful patient selection and further research into the mechanisms underlying PSMA uptake in ACC are essential before clinical application of ^177^Lu-PSMA therapy.

## Supplementary Information

Below is the link to the electronic supplementary material.

Supplementary Material 1 (DOCX 47.0 KB)

## Data Availability

Contact the corresponding author for data requests.
