# Supplementary material for: Clinical Characteristics Associated with High [68Ga]Ga-PSMA-11 Uptake and Preliminary Therapeutic Outcomes in Patients with Metastatic Adenoid Cystic Carcinoma
Source: Nucl Med Mol Imaging. 2025 Oct 23;60(1):21–8. doi: 10.1007/s13139-025-00955-9 (PMC12882889; doi:10.1007/s13139-025-00955-9)
Supplement: Supplementary file 1 — Supplementary Material 1 (DOCX 47.0 KB) [file 13139_2025_955_MOESM1_ESM.docx]

**Supplementary Data**

**Clinical Characteristics Associated with High [^68^Ga]Ga-PSMA-11 uptake in Metastatic Adenoid Cystic Carcinoma**

**Supplementary Figure 1.** Comparison of PSMA uptake in liver, salivary gland, and blood pool between high and low PSMA group

**Supplementary Table 1.** Patient characteristics

| No. | Gender | Age | Primary tumor location | Predominant type | Initial stage | Local therapy | Chemotherapy | Ongoing treatment | Time from diagnosis (mo.) | Time from recurrence (mo.) | Metastases site | Median tumor SUV_max_ |
| --- | --- | --- | --- | --- | --- | --- | --- | --- | --- | --- | --- | --- |
| 1 | F | 61 | Trachea | N/A | 1 | Done | Done | Active surveillance | 64.3 | 64.3 | Lung | 6.1 |
| 2 | F | 50 | Submandibular gland | Tubular | 4c | Done | Not done | Active surveillance | 11.6 | 11.6 | Lung, bone, liver | 2.2 |
| 3 | F | 73 | Submandibular gland | N/A | 4c | Done | Done | Active surveillance | 19.6 | 19.6 | Lung, bone | 4.0 |
| 4 | M | 55 | Palate | Tubular | 1 | Done | Not done | Active surveillance | 69.8 | 43.4 | Lung | 6.8 |
| 5 | M | 41 | Nasopharynx | N/A | N/A | Done | Done | Cyclophosphamide, doxorubicin, cisplatin | 227.2 | 2.0 | Pleura | 3.0 |
| 6 | M | 61 | Nasopharynx | Cribriform | 1 | Done | Done | Cisplatin, vinorelbine | 82.5 | 82.5 | Lung, bone | 6.9 |
| 7 | M | 68 | Parotid gland | Solid | 4c | Done | Not done | Active surveillance | 43.1 | 43.1 | Lung, liver | 2.8 |
| 8 | F | 66 | Trachea | N/A | N/A | Done | Not done | Active surveillance | 57.6 | 15.6 | Lung, bone | 2.1 |
| 9 | F | 56 | Submandibular gland | Cribriform | 4a | Done | Not done | Active surveillance | 11.0 | 11.0 | Lung | 1.5 |
| 10 | M | 50 | Maxillary sinus | Cribriform | 4a | Done | Not done | Active surveillance | 19.6 | 5.9 | Lung | 2.4 |
| 11 | M | 51 | Submandibular gland | N/A | 1 | Done | Done | Lenvatinib | 136.3 | 30.8 | Lung | 4.8 |
| 12 | M | 66 | Parotid gland | N/A | N/A | Done | Not done | Active surveillance | 95.8 | 46.8 | Lung, bone | 3.2 |
| 13 | F | 73 | Palate | N/A | 3 | Done | Not done | Active surveillance | 157.6 | 136.7 | Lung | 7.0 |
| 14 | M | 67 | Submandibular gland | Cribriform | 1 | Done | Done | Lenvatinib | 55.1 | 46.5 | Lung | 2.5 |
| 15 | F | 67 | Tongue | N/A | 1 | Done | Not done | Active surveillance | 134.5 | 41.9 | Lung, pleura | 3.9 |
| 16 | F | 64 | Floor of mouth | Cribriform | 1 | Done | Not done | Active surveillance | 76.8 | 30.8 | Lung | 7.8 |
| 17 | M | 59 | Submandibular gland | Tubular, cribriform | 2 | Done | Not done | Active surveillance | 6.7 | 6.7 | Lung, bone | 3.4 |
| 18 | F | 56 | Palate | Solid | 4b | Done | Not done | Active surveillance | 7.2 | 1.1 | Bone | 6.1 |
| 19 | M | 64 | Submandibular gland | Cribriform | 1 | Done | Not done | Active surveillance | 145.8 | 29.1 | Pleura | 7.4 |
| 20 | M | 43 | Parotid gland | Tubular | 4b | Done | Not done | Active surveillance | 67.8 | 67.8 | Bone | 4.7 |
| 21 | F | 42 | Lip | Solid | 1 | Done | Done | Active surveillance | 53.0 | 46.9 | Lung, bone, brain | 5.4 |
| 22 | F | 55 | Palate | N/A | 4a | Done | Not done | Active surveillance | 90.5 | 53.8 | Lung, bone, liver | 4.1 |
| 23 | F | 66 | Parotid gland | N/A | N/A | Done | Done | Active surveillance | 432.7 | 40.0 | Bone, pleura | 8.1 |
| 24 | M | 65 | Submandibular gland | Solid | 2 | Done | Done | Active surveillance | 31.8 | 8.2 | Lung, brain | 7.7 |
| 25 | F | 63 | Tonsil | N/A | 1 | Done | Not done | Active surveillance | 58.8 | 30.4 | Lung | 2.7 |
| 26 | F | 50 | Parotid gland | N/A | N/A | Done | Done | Active surveillance | 204.8 | 204.8 | Pleura | 5.2 |
| 27 | M | 33 | Submandibular gland | Solid | 1 | Done | Not done | Active surveillance | 39.9 | 9.5 | Lung, bone, liver, lymph node | 6.5 |
| 28 | F | 45 | Tongue | Cribriform | 4c | Done | Done | Active surveillance | 64.3 | 64.3 | Lung | 1.9 |
| 29 | F | 54 | Parotid gland | Cribriform | 4a | Done | Not done | Active surveillance | 34.6 | 27.8 | Lung | 2.9 |
| 30 | M | 72 | Maxillary sinus | Tubular, cribriform | 4a | Done | Not done | Active surveillance | 209.3 | 4.2 | Lung, bone | 3.5 |

**Supplementary Table 2.** Patient received ^177^Lu-DGUL therapy characteristics

| No. | Gender | Age | Primary tumor location | Predominant type | Initial stage | Metstases site | Treatment Cycles | Normal liver SUV_mean_ | Highest Tumor SUV_peak_ | Time to progression (days) | | | Treatment response |
| --- | --- | --- | --- | --- | --- | --- | --- | --- | --- | --- | --- | --- | --- |
| 1 | M | 55 | Palate | Tubular | 1 | Lung | 2 | 3.8 | 12.1 | | 86 | PD | |
| 2 | F | 42 | Lip | Solid | 1 | Lung, bone, brain | 2 | 2.7 | 7.1 | | 104 | SD | |
| 3 | F | 66 | Parotid gland | N/A | N/A | Bone, pleura | 1 | 2.8 | 10.0 | | N/A | N/A | |
| 4 | M | 33 | Submandibular gland | Solid | 1 | Lung, bone, liver, lymph node | 2 | 4.2 | 8.7 | | 86 | PD | |
| 5 | F | 45 | Tongue | Cribriform | 4c | Lung | 2 | 1.0 | 2.7 | | 100 | SD | |
| 6 | M | 70 | Submandibular gland | N/A | 2 | Lung | 1 | 1.4 | 4.3 | | N/A | N/A | |
| 7 | M | 72 | Maxillary sinus | Tubular, cribriform | 4a | Lung, bone | 1 | 3.7 | 8.2 | | N/A | N/A | |

N/A: Non-applicable. PD: Progressed disease. SD: Stable disease
